# The interplay of mutagenesis and extracellular DNA: a new chapter in urothelial cancer research

**DOI:** 10.1186/s43556-025-00295-2

**Published:** 2025-07-24

**Authors:** Xuan Zhang, Yirong Li, Xinghua Long

**Affiliations:** Department of Laboratory Medicine, Zhongnan Hospital of Wuhan University, Wuhan, China

In a recent pioneering study published in Nature [1], Duy D. Nguyen and colleagues present a transformative investigation into the synergistic interplay between mutagenesis and extracellular DNA (ecDNA) in shaping the evolutionary dynamics of urothelial carcinoma (UC). Currently, despite advances in oncology, advanced UC remains an incurable disease with genetic heterogeneity. This study delineates pivotal molecular mechanisms underlying tumorigenesis and therapeutic resistance in UC, offering a novel perspective on cancer biology (Fig. 1).

**Fig. 1:**
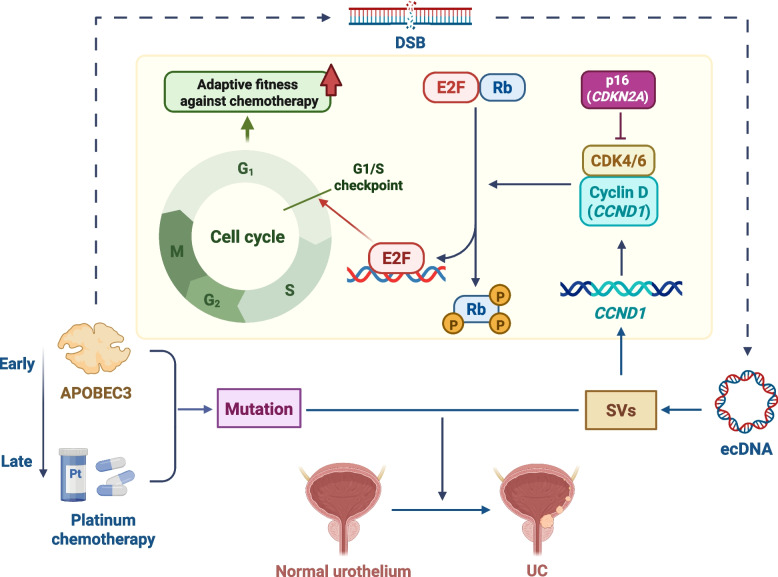
Fundamental mechanisms underlying the evolution and therapeutic resistance in UC. Early APOBEC3-induced and late chemotherapy-induced mutations as well as ecDNA-forming structural variations (SVs) drive UC evolution. APOBEC3-induced double-strand breaks(DSBs) indirectly play a potential role in ecDNA biogenesis. The p16–cyclin D1–CDK4/6–RB1 pathway is activated by ecDNA-driven *CCND1* amplification, ultimately enhancing cell proliferation and adaptability to chemotherapy. Moreover, *CDKN2A* homozygous deletions will promote cancer progression. (created in BioRender.com [https://biorender.com])

Through rigorous experimentation, the authors elucidated that ecDNA—circular DNA structures located outside the chromosomal genome—magnifies oncogene expression in cancer cells through oncogene amplification, fostering aggressive tumor phenotypes. By performing long-read whole-genome sequencing (WGS), they elegantly mapped the structural and genetic evolution of ecDNA in UC, uncovering its role in tumorigenesis and therapeutic resistance.

ecDNA is present in nearly half of human cancers with a frequency varied by tumor type, but rarely found in normal cells [2]. Oncogenes are amplified most commonly in ecDNA, driving elevated oncogene copy number and fostering intratumoral heterogeneity. A recent study suggested that oncogene *CCND1* was amplified in breast cancer through ecDNA formed by translocations [3].The amplification mechanism allows cancers to evade therapies targeting oncogenes maintained on ecDNA.

Previous studies have indicated that the apolipoprotein B mRNA-editing enzyme catalytic subunit (APOBEC) is particularly prominent in advanced UC and proposed that APOBEC3A is the main driver of APOBEC mutagenesis [4]. A key aspect of the research is the examination of the role of the APOBEC3 family. The APOBEC3 family of cytosine deaminases can lead to the replacement of cytosine with thymine or guanine in the genomes of many human cancers, generating somatic mutation signatures, particularly in UC [1, 4]. The data demonstrated that these enzymes played a dual role, promoting not only somatic mutation but also ecDNA evolution.

Platinum-based chemotherapy remains the primary first-line treatment for most patients with advanced UC. For cisplatin-ineligible patients, combination therapy with immune checkpoint inhibitors and antibody–drug conjugates has obtained accelerated FDA approval as first-line therapy [5]. Nguyen et al. demonstrate a mechanistic interplay between ecDNA and mutagenic processes in UC. Through systematic comparison of APOBEC3 and chemotherapy-induced SVs in ecDNA, the authors described a spatiotemporal hierarchy and mechanisms of ecDNA biogenesis. The analysis clearly revealed that APOBEC3 and chemotherapy collaborated synergistically to drive ecDNA genesis via temporally distinct and mechanistically divergent pathways. During the early stages of tumor evolution, APOBEC3 could promote ecDNA genesis through inducing DSBs. Following therapeutic intervention, chemotherapy-induced mutations further accelerated ecDNA evolution, amplifying intratumoral heterogeneity and fostering acquired drug resistance.

By constructing the phylogenetic tree, the authors systematically mapped the temporal landscape of mutagenesis during tumor evolution in ecDNA-derived SVs. Their analysis uncovered a distinct chronological stratification: APOBEC3-associated mutations arose as clonal events during initial tumorigenesis, while chemotherapy-induced mutations predominantly accumulated as late-stage, subclonal alterations. These findings suggested dual therapeutic opportunities—early intervention via APOBEC3 inhibition to prevent oncogenic initiation and adaptive chemotherapy scheduling to circumvent ecDNA-mediated resistance mechanisms.

Cyclin D1, encoded by *CCND1*, serves as a pivotal regulator of G1/S phase progression by modulating the activity of cyclin-dependent kinases 4 and 6 (CDK4/6) [1]. Dysregulated *CCND1* expression disrupts cell cycle checkpoint control, leading to aberrant proliferation. By analyzing high-frequency, high-copy-number ecDNA amplification sites *CCND1*, the researchers demonstrated UC tumors with ecDNA-mediated *CCND1* amplification activate the p16–cyclin D1–CDK4/6–RB1 pathway, which underscores the importance of ecDNA in driving oncogenic processes. Meanwhile, the homozygous deletion of *CDKN2A* (encoding the tumor suppressor p14 and p16) was more common in UC with ecDNA-mediated *CCND1* amplification. Similarly, it is worth noting that p14 serves a pivotal function in tumor suppression, and its loss may trigger cancer development through the p53 pathway. Notably, *CDKN2A* heterozygous deletion is significantly more prevalent than *CCND1* amplification in UC, suggesting that tumorigenesis often occurs through *CDKN2A* loss, thereby relieving the inhibition of CDK4/6. However, Nguyen et al.'s study shows that in UC samples with *CCND1* amplification via ecDNA, 87% exhibit concurrent *CDKN2A* loss. This indicates that in certain cases, *CCND1* amplification does not necessarily coincide with *CDKN2A* loss, implying that these two mechanisms may independently contribute to cancer progression. Furthermore, these ecDNA events persist and become highly complex and incorporate new DNA fragments after treatment, suggesting that they drive cancer resistance to therapy.

In the study of *CCND1* ecDNA driving chemotherapy resistance, the authors found that *CCND1* ecDNA significantly enhances tumor cell adaptation to chemotherapy by driving cell cycle progression through activation of the downstream E2F signaling pathway, demonstrating the fitness advantage of *CCND1* ecDNA in driving resistance to chemotherapy.

A standout feature of the research is its interdisciplinary approach, combining genomics, molecular biology and advanced computational analysis. The authors employed cutting-edge sequencing technologies to unravel the structure and functional roles of ecDNA in urothelial cancer, providing a comprehensive molecular atlas that could serve as a valuable reference for future cancer studies. The technological advance allows for a more comprehensive analysis of genomic alterations and their functional consequences. Their computational framework identified mutation hotspots and gene amplifications in ecDNA, a methodological innovation with broad applications for investigating other cancer types as well.

Although the implications of the study are promising, certain challenges remain. First, due to the small sample size, their study fails to draw a definitive correlation between mutation and structural variation events and clinical outcomes. In the future, WGS studies with prospective clinical annotation are needed in a larger population of patients with advanced UC tumors. Second, given that p14 encoded by *CDKN2A* gene plays a critical role in p53 dynamics, it is necessary to conduct further exploration of p14. Finally, the exact mechanism of ecDNA formation is not fully clear and targeted therapies against ecDNA are still in their infancy, with technical and ethical challenges that need to be addressed in future work. Nevertheless, Nguyen et al.’s research represents a crucial step toward harnessing ecDNA as both a biomarker and a therapeutic target in UC.

Overall, the study provides a transformative perspective on cancer evolution by targeting ecDNA as a key driver of UC tumor progression and diversity. By elucidating the interactions between ecDNA and mutagenesis, the study provides a solid foundation for innovative therapeutic approaches. The article is not only a testament to the role of ecDNA in cancer biology, but also a call to action for further research to unlock its therapeutic potential.

## Data Availability

Not applicable.
